# Deletion of *Pr130* Interrupts Cardiac Development in Zebrafish

**DOI:** 10.3390/ijms17111746

**Published:** 2016-11-11

**Authors:** Jie Yang, Zuhua Li, Xuedong Gan, Gang Zhai, Jiajia Gao, Chenling Xiong, Xueping Qiu, Xuebin Wang, Zhan Yin, Fang Zheng

**Affiliations:** 1 Center for Gene Diagnosis, Zhongnan Hospital of Wuhan University, Wuhan 430071, China; manbuyunhailzh@163.com (J.Y.); lzu@whu.edu.cn (Z.L.); flower198901@163.com (J.G.); perkinshine@yeah.net (C.X.); happytime19881@yeah.net (X.Q.); xbwang2013@whu.edu.cn (X.W.); 2 Department of Cardiology, Zhongnan Hospital of Wuhan University, Wuhan 430071, China; sunshine8789@yeah.net; 3 Key Laboratory of Aquatic Biodiversity and Conservation of Chinese Academy of Sciences, Institute of Hydrobiology, Chinese Academy of Sciences, Wuhan 430072, China; zhaigang19@126.com

**Keywords:** CRISPR-Cas9, *pr130*, cardiac development, zebrafish

## Abstract

Protein phosphatase 2 regulatory subunit B, alpha (*PPP2R3A*), a regulatory subunit of protein phosphatase 2A (PP2A), is a major serine/threonine phosphatase that regulates crucial function in development and growth. Previous research has implied that *PPP2R3A* was involved in heart failure, and PR130, the largest transcription of *PPP2R3A*, functioning in the calcium release of sarcoplasmic reticulum (SR), plays an important role in the excitation-contraction (EC) coupling. To obtain a better understanding of PR130 functions in myocardium and cardiac development, two *pr130*-deletion zebrafish lines were generated using clustered regularly interspaced short palindromic repeats (CRISPR)/CRISPR-associated proteins (Cas) system. *Pr130*-knockout zebrafish exhibited cardiac looping defects and decreased cardiac function (decreased fractional area and fractional shortening). Hematoxylin and eosin (H&E) staining demonstrated reduced cardiomyocytes. Subsequent transmission electron microscopy revealed that the bright and dark bands were narrowed and blurred, the Z- and M-lines were fogged, and the gaps between longitudinal myocardial fibers were increased. Additionally, increased apoptosis was observed in cardiomyocyte in *pr130*-knockout zebrafish compared to wild-type (WT). Taken together, our results suggest that *pr130* is required for normal myocardium formation and efficient cardiac contractile function.

## 1. Introduction

Protein phosphorylation is tightly synchronized by balanced kinases and phosphatases. Defects in protein phosphorylation will result in a variety of cardiac diseases, including heart failure and atrial fibrillation [1,2]. PP2A is a remarkably conserved serine/threonine phosphatase in eukaryotes and is involved in a wide range of normal biologic processes such as growth, development, cell apoptosis, and signal transduction [3,4,5]. Notably, PP2A is also one of the major phosphatases in the heart, regulating membrane excitability and cardiac excitation-contraction through dephosphorylating an array of ion channels and cardiac proteins [6].

PP2A core enzyme, composed of a scaffolding A subunit and a catalytic C subunit, binds with a variable regulatory B subunit to form a heterotrimeric complex, of which the subunit B dominates the specificity of substrate, holoenzyme localization, and activity [7]. So far, at least four families of PP2A regulatory B subunit have been identified. *PPP2R3A* that belongs to the regulatory subunit B family of PP2A has been reported to be located on both Z- and M-lines in myocardium and to be involved in heart failure [7]. PR130, the largest transcript of *PPP2R3A*, is highly expressed and exhibits a striated expression pattern in the heart [3]. PR130 was reported to be involved in the calcium release of sarcoplasmic reticulum (SR) as a binding target of Ryanodine receptor 2 (RyR2), which was closely related to myocardium excitation-contraction (EC) coupling [6], suggesting that PR130 may play essential roles in cardiac contractile function. Moreover, the expression of mPR130 (mouse PR130) was increased in embryonic development proceeds [3]. Additionally, depletion of Xpr130 (Xenopus PR130) caused development defects [8]. PR130 was also a positive modulator of wnt/β-canonical signaling, which is essential for cardiogenesis [8,9]. Taken together, these studies suggest that PR130 may play a critical role in cardiac development.

Zebrafish represent an excellent vertebrate model and have been widely employed to study heart development [10]. CRISPR/Cas system served as a high efficient targeted genome mutagenesis tool, which had been applied to cultured human cells, mice, yeast, as well as zebrafish [11]. In order to investigate the role of PR130 in the heart, we generated two heritable and precise zebrafish lines with endogenous *pr130*-knockout using CRISPR/Cas system to provide a better understanding of PR130 functions in myocardium and heart development.

## 2. Results

### 2.1. Expression Patterns of Pr130 in Zebrafish

**Figure 1:**
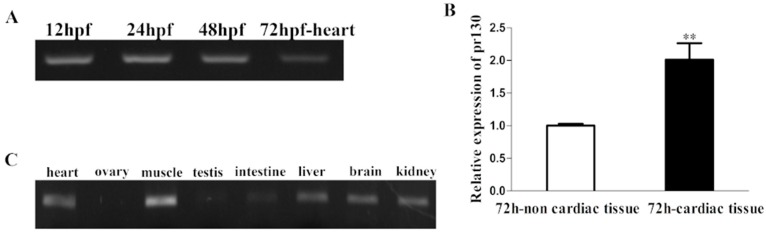
Expression patterns of *pr130* in zebrafish. (**A**) Expression of *pr130* at different stages in whole fish during zebrafish embryogenesis and in 72 hpf-heart; (**B**) Relative expression of *pr130* in non-cardiac tissue and cardiac tissue of 72 hpf embryos was identified using real-time reverse transcription-polymerase chain reaction (RT-PCR); (**C**) The expression of *pr130* in different tissues of adult zebrafish. Data represent the mean ± SD. **, *p* < 0.01.

To study the in vivo role of *pr130* in zebrafish cardiac development, we first analyzed the homology of PR130 and *pr130* between human and zebrafish using BLAST analysis. The putative zebrafish *pr130* is located on chromosome 2 and encodes a protein of 1192 amino acids, which has a 67% homology to human PR130. The expression profile of *pr130^-/-^* in zebrafish during embryogenesis and in different adult organs was identified, the results showed that *pr130* could be detected in zebrafish embryos from 12 ho post-fertilization (hpf) stage to 48 hpf stage, 72 hpf-heart (Figure 1A), and in adult heart, muscle, testis, intestine, liver, brain, and kidney (Figure 1C), as revealed by reverse transcription-polymerase chain reaction (RT-PCR). Furthermore, comparing with 72h-non-cardiac tissues, 72 cardiac tissues had higher levels of *pr130* expression (Figure 1B). The expression patterns of a gene usually provide important clues to its function. High expression of *pr130* in heart hints that *pr130* may contribute to cardiac function.

### 2.2. Deletion of Pr130 Results in Lower PP2A Activity

**Figure 2:**
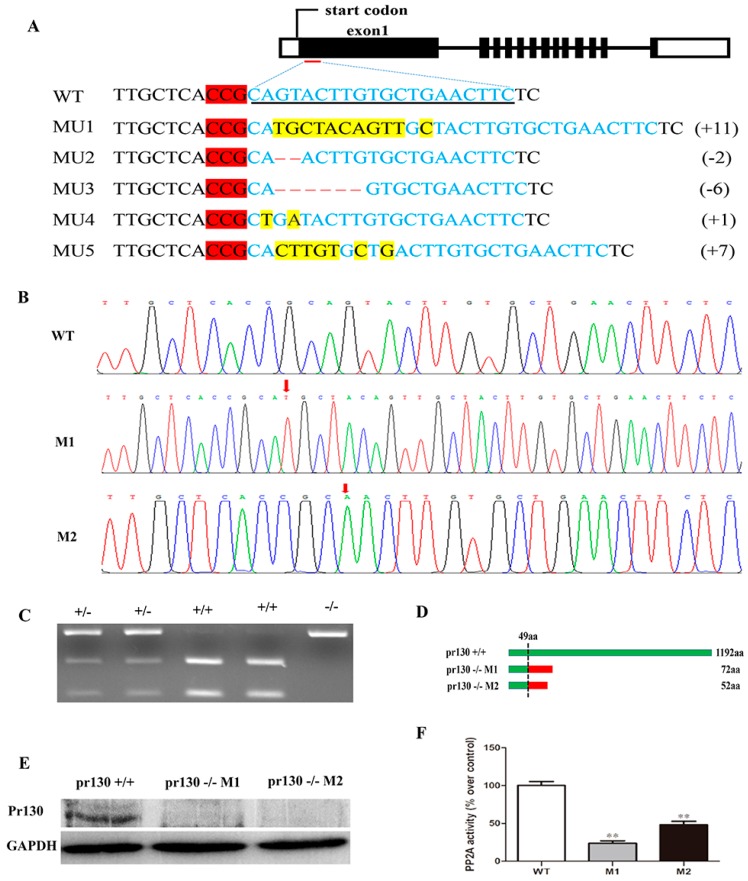
Schematic diagram of *pr130* knock-out in zebrafish using CRISPR/Cas9 system and analysis of PP2A activity. (**A**) Sequence of target site is underlined with black line and is shown in blue font in wild-type (WT) and protosacer adjacent motif (PAM) highlighted in red. Deletions and insertions are indicated by dashes and yellow highlight, respectively. Changed nucleotides are shown on the right side, “+” indicates insertion and “-” indicates deletion; (**B**) The sequences of WT, M1 and M2, the regions of insertion (M1) and deletion (M2) are indicated by red arrows; (**C**) A representative genotyping of *ScaI* digestion of PCR products amplified from genomic DNA, the products amplified from WT samples could be digested completely, whereas those of the homozygous mutants could not be digested, and those of the heterozygous mutants could be partially digested. +/+ indicates WT, (+/-) indicates heterozygous mutants, (-/-) indicates homozygous mutants; (**D**) The predicted amino acids of Pr130, in which showed a truncated protein, the first 49 aa (**green**) are identical to those of the WT Pr130 protein, which contains 23 (M1) or 3 (M2) miscoding amino acids (**red**); (**E**) Western blotting analysis of Pr130 protein; (**F**) PP2A activity was determined in cardiac extracts of WT and mutants. Data represent the mean ± SD. The symbols ** in the bar chart represent significant differences (*p* < 0.01).

Zebrafish *pr130* knockout lines were generated utilizing CRISPR/Cas9 system, and mutants were selected using restriction fragment length polymorphism (RFLP) with *Sca*I enzyme digestion (Figure 2C) and confirmed using Sanger sequencing (Figure 2B). In F0 founders, five independent mutation lines (Figure 2A) were obtained, four of which contained frameshift mutations, and the mutation rate was 83.3%. Due to the limitation of the number and proportion of male and female in F0 mutants, we chose only two mutant lines in this experiment, named M1 (MU1: 11 nucleotides insertion) and M2 (MU2: two nucleotides deletion) (Figure 2A), which result in premature termination of the mutant (Figure 2D). The sequences of M1 and M2 are shown in Figure 2B. As expected, Pr130 protein was undetectable in the mutants as evidenced by western blotting (Figure 2E).

The B regulatory subunits adjusted the activity and the specific intracellular location of PP2A. Inhibition of PR130 by shRNA could lead to decreased activity of PP2A [12], however inhibition of B56α induced enhanced activity of PP2A [13]. To test whether PP2A activity was altered in *pr130* knockout zebrafish, the PP2A activity of the heart was measured. The result revealed that PP2A activity was decreased in mutant hearts compared to wild-type (WT) (Figure 2F).

### 2.3. Increased Mortality and Defective Cardiac Development in Pr130^-/-^ Zebrafish

**Figure 3:**
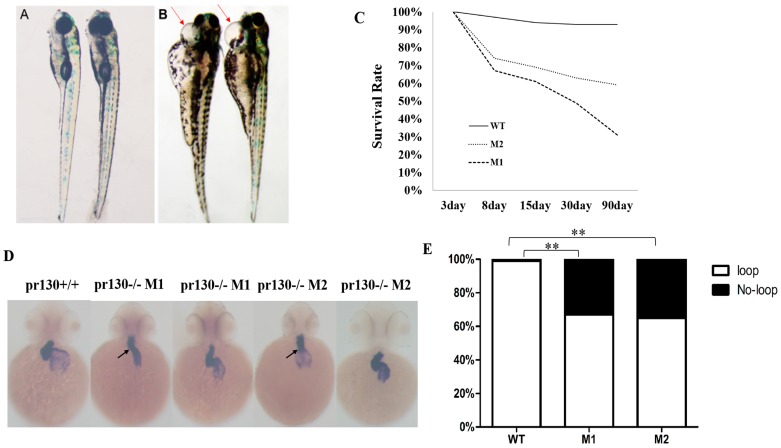
Effects of *pr130* deletion on mortality and cardiac development. (**A**,**B**) *pr130^-/-^* embryos develop pericardial edema (**arrow**). **A**, WT; **B**, *pr130^-/-^*; (**C**) Survival curves; (**D**) Whole-mount in situ hybridization (WISH) using the heart probe *cmlc2*. Mutants showed a higher proportion of no-looping (**arrows**) compared with WT which showed normal heart looping; (**E**) Percentage of abnormal and normal heart looping. *n* = 100 embryos analyzed per panel. The symbols ** in the bar chart represent significant differences (*p* < 0.01).

Xpr130 is suggested to be involved in development and growth of Xenopus [8]. To investigate whether *pr130* had a similar role in growth for zebrafish, the survival rate was calculated. We analyzed the survival rate from the fourth day. The survival rate of *pr130^-/-^* zebrafish was statistically significantly lower than controls (Figure 3C). *Pr130^-/-^* zebrafish showed a peak of mortality rate of 30% or so at seven days post-fertilization (dpf), while 63% of the dying zebrafish exhibited severe pericardial edema (Figure 3A,B) and a faint heartbeat. These data suggested that *pr130* might contribute to the regulation of zebrafish cardiac development.

At 48 hpf, WT zebrafish hearts underwent an S-looping process [14,15]. We monitored the heart development with the cardiac-specific marker *cmlc2* by whole-mount in situ hybridization (WISH). *Pr130^-/-^* zebrafish displayed a reduction in the level of *cmlc2* expression in atrium and ventricle and a higher proportion of abnormal shape loops. About 33% of the M1 embryos and 35% of the M2 embryos showed un-looped shape, in contrast, 99% of the WT embryos showed an S-loop (Figure 3D,E). The first seven days were critical for the morphological development and structure formation of the zebrafish hearts [15], thus, it was possible that *pr130*-knockout led to death due to cardiac development defects.

**Figure 4:**
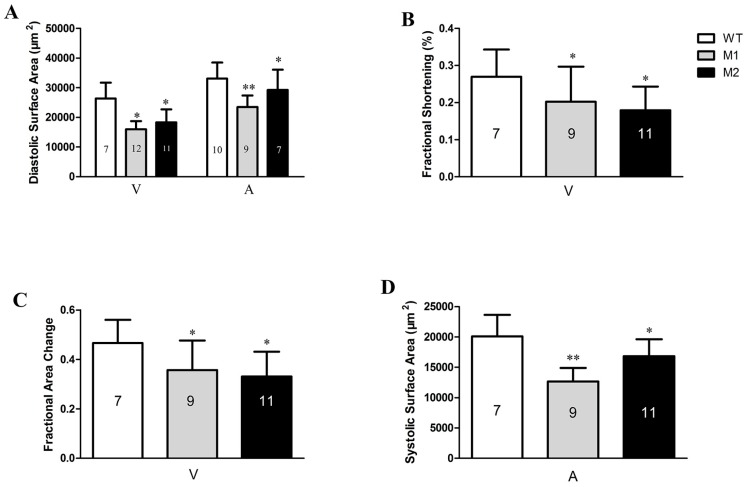
*Pr130* deletion causes defective function of heart. (**A**) The diastolic surface areas of ventricular and atrial; (**B**) Fractional shortening of ventricular; (**C**) Fractional area change of ventricular; (**D**) The systolic surface areas of atrial. V, ventricular; A, atrial. Data represent the mean ± SD. The symbols * and ** in the bar chart represent significant differences (*p* < 0.05 or *p* < 0.01). The numbers of zebrafish are indicated in the columns.

To further elucidate the impact on cardiac function, we quantitatively analyzed it by optical heartbeat analysis. Statistically, *pr130^-/-^* embryos displayed a significant reduction in diastolic surface areas, fraction shortening, and fractional area changes of ventricle, as well as decreased diastolic surface areas and systolic surface areas in atrium (Figure 4).

### 2.4. Altered Cardiac Structure and the Decrease of Cardiomyocytes in Pr130^-/-^ Zebrafish

**Figure 5:**
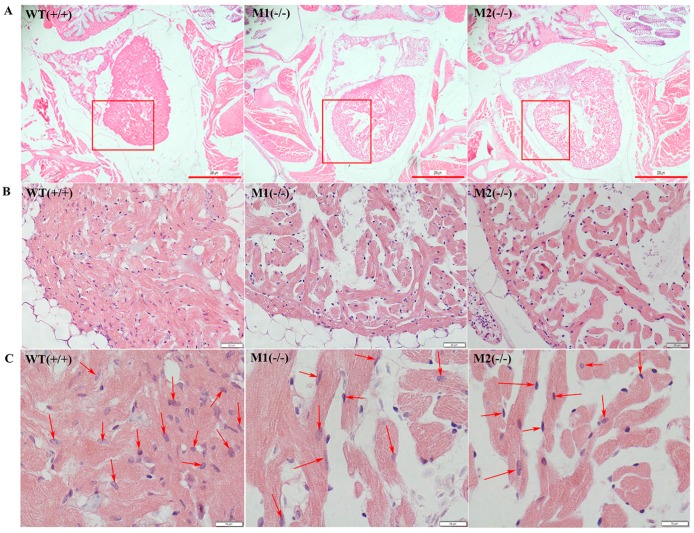

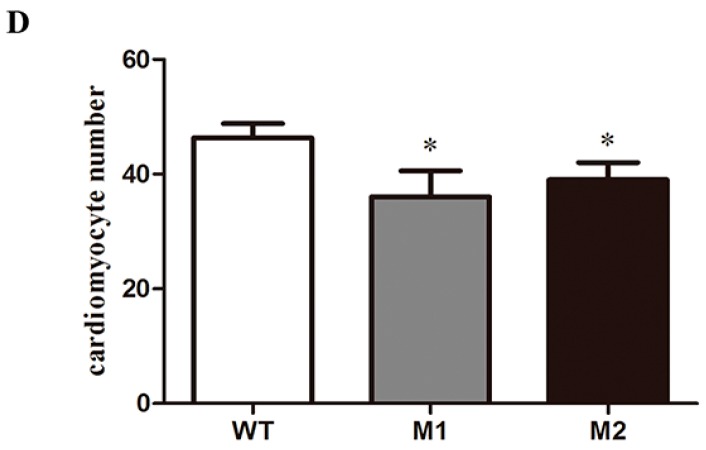
Decreased cardiomyocytes in *pr130^-/-^* zebrafish. (**A**) Representative images of H&E staining of adult heart tissue at 40× magnification. Scale bars, 200 µm; (**B**) Higher magnification of the boxed regions (400× magnification). The heart tissues of WT, M1, and M2 were from the same region of heart. Scale bars, 20 µm; (**C**) *Pr130^-/-^* exhibited reduced cardiomyocytes. Red arrows indicate the myocardium nucleus. Scale bars, 10 µm; (**D**) The cardiomyocytes in heart tissues of controls and *pr130^-/-^* adult zebrafish were quantified and presented in the bar graph. We calculated the number of cardiomyocytes in each of four chosen fields per fish at a magnification of 1000×. *n* = 16 hearts analyzed per panel. Data represent the mean ± SD. *, *p* < 0.05.

**Figure 6:**
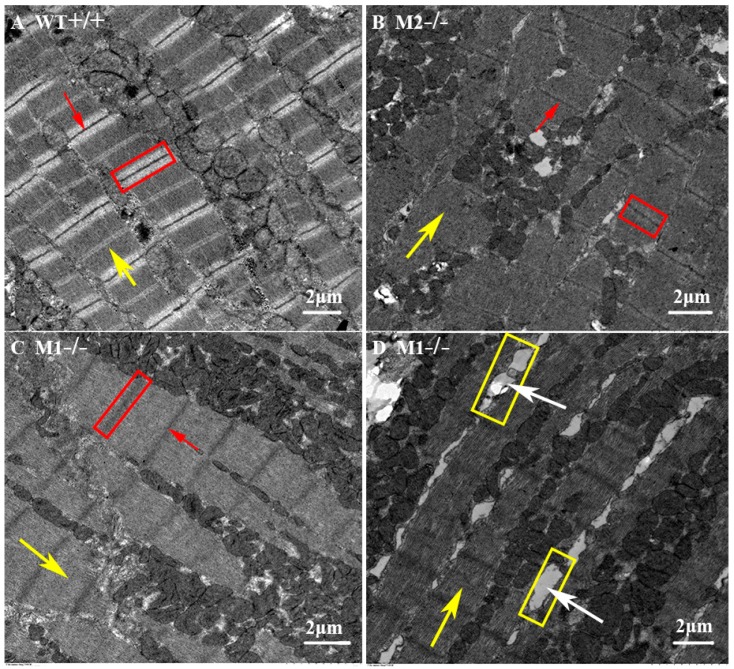
Ultrastructure of adult heart tissues observed using transmission electron microscopy. *Pr130^-/-^* zebrafish displayed disordered myocardium. In *pr130^-/-^* heart, the boundary of bright band (**red boxes**), M line (**yellow arrows**), and Z line (**red arrows**) were not clear. In some cases the gaps between some longitudinal myocardial fibers increased (**yellow boxes** and **white arrows**).

To further study the consequence of *pr130*-knockout on heart development, we analyzed the cardiac structure of adult zebrafish using H&E staining and transmission electron microscopy. H&E staining of cardiac sections revealed that the numbers of cardiomyocytes from the same region of heart were decreased in the mutants compared with WT (Figure 5). Transmission electron microscopy demonstrated that *pr130*^-/-^ displayed disorganized ultrastructure and narrowed bright and dark bands. The boundaries of bright and dark bands became less clear along with the Z- and M-lines; gaps between some longitudinal myocardial fibers were increased (Figure 6); and Z- and M-lines became blurred (Figure 6), which was coincident with the PPP2R3A location on Z- and M-lines [7].

### 2.5. Increased Apoptosis Caused the Reduction of Cardiomyocytes

**Figure 7:**
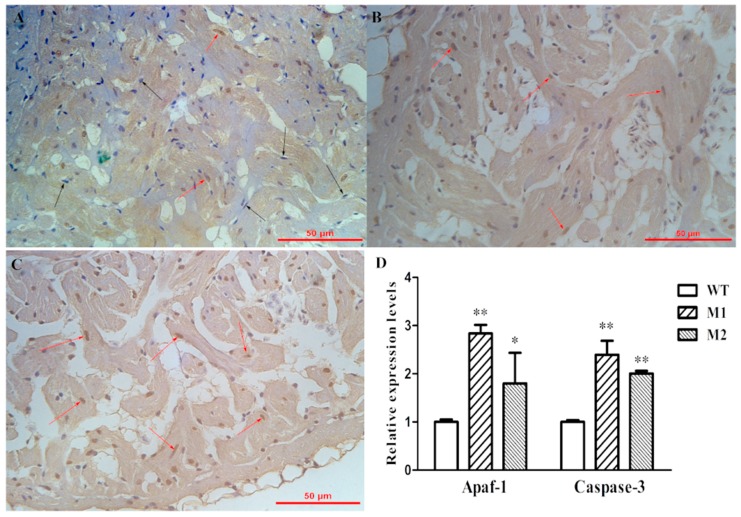
Increased apoptosis was observed in *pr130^-/-^*. (**A**–**C**) Representative images of Tunel staining sections of adult heart tissue of WT (**A**), M1 (**B**), and M2 (**C**). Black arrows indicate normal cells (**blue**), red arrows indicate apoptosis cells (**brown**); (**D**) Expression levels of apoptosis-associated genes from adult heart tissues. Data represent the mean ± SD. The symbols * and ** in the bar chart represent significant differences (*p* < 0.05 or *p* < 0.01).

Previous research had revealed that PP2A is an important modulator in apoptosis signaling [5]. We hypothesized that reduction of cardiomyocyte numbers in the heart may be a result of apoptosis. As such, we performed terminal deoxynucleotidyl transferase-mediated dUTP nick end labeling (Tunel) staining on adult hearts, which revealed that the apoptosis level in *pr130^-/-^* hearts was increased (Figure 7A–C). We further analyzed the expression of apoptosis related genes, including *Mdm2*, *Bax*, *Bcl*-*2*, *Puma*, *Apaf*-*1*, *Caspase*-*9*, and *Caspase*-*3* using quantitative polymerase chain reaction (qPCR) [16]. Among them, only *Apaf*-*1* and *Caspase*-*3* mRNA levels were significantly increased in *pr130^-/-^* compared to WT (Figure 7D). The mRNA expression of other apoptosis genes was not increased in *pr130^-/-^* compared to WT. As such, we concluded that *pr130* might lead to a reduction of cardiomyoctyes through increased apoptosis via *Caspase*-*3* and *Apaf*-*1* pathway.

## 3. Discussion

To study the role of *pr130* in heart development, we utilized the CRISPR/Cas9 system to establish two stable *pr130*-knockout mutants. *Pr130^-/-^* zebrafish exhibited the following characteristics: (i) Pr130 protein was undetectable in five dpf embryos by western blot and PP2A activity of adult heart tissues were decreased; (ii) a higher mortality rate and cardiac development defects including higher proportion of pericardial edema and cardiac looping defects were observed; (iii) the structure of myocardium was disorganized and the myocytes were decreased; (iv) impaired cardiac function of *pr130^-/-^* zebrafish was detected; (v) increased apoptosis in *pr130* mutants as evidenced by Tunel staining. Thus, we concluded that *pr130* displays important roles in cardiac development in zebrafish.

Decreased PP2A activity in the heart was likely caused by *pr130*-knockout, which when induced by unstable truncated Pr130 premature termination. This was coincident with the undetected Pr130 using western blot (Figure 2E). The excessive expression of *pr130* in the heart further indicated that *pr130* was involved in the location of PP2A in the heart. As such, we hypothesized that *pr130*-knockout interrupted the heart development probably via defected dephosphorylation on cardiac contractile proteins due to decreased PP2A activity in *pr130^-/-^* zebrafish heart. Notably, dephosphorylation of RyR2 is controlled by PP2A via binding with PR130 [6]. Certainly, the heart phenotype of *pr130^-/-^* could also be induced by general deficient dephosphorylation of PP2A holoenzyme instead of a specific subunit, because PP2A activity is involved in the dephosphorylation of multiple ion channels and cardiac contractile proteins of myocardium [1,6,17]. Actually, other cases have been reported where the lack of some other subunit exhibited similar heart phenotypes with *pr130^-/-^*. For example, transgenic mice expressing mutant A subunits of PP2A that cannot bind to B subunits had decreased fractional shortening, which was the same as what we observed in *pr130^-/-^* (Figure 4B) [18]. B56γ, a regulatory subunit of PP2A, was reported to play an essential role in heart development and lack of B56γ in a transgenic mouse resulted in a decrease in the number of cardiomyocytes, which was similar to what we observed in *pr130^-/-^* zebrafish too (Figure 5C,D) [19].

The influence on PP2A activity due to the deletion of a PP2A regulatory subunit is debated and not well understood. Our study demonstrated that knockout of *pr130* leads to decreasing cardiac PP2A activity. A previous study reported that PR130 contains a conserved region with two A subunit binding domains (ASBD) and two EF-hand motifs, which is important for binding to PP2A A subunits [3]. Contrary to our results, the deficiency of B56α (another regulatory of PP2A), which is an autoinhibitor of cardiac PP2A activity, resulted in increased PP2A activity [13]. However, some studies revealed that B56α deficiency may decrease PP2A activity too, and overexpression of B56α enhanced PP2A activity in the heart [13,20]. It is possible that different regulatory subunits of PP2A have different influences in PP2A activity. Moreover, the regulatory subunits may have contradicting roles in regulating cardiac PP2A activity, based on the myocardium microenvironment.

Our study found that the apoptosis signal was increased in *pr130^-/-^* myocardium (Figure 7), although the exact mechanism needs further research. However, these results were contradictory to the references that noted that increased PP2A activity leads to apoptosis and inhibition of PP2A by okadaic acid (a specific PP2A inhibitor) may prevent apoptosis [5,21]. In addition to the role of PP2A in apoptosis, *pr130* may contribute directly to apoptosis. Interestingly, a previous study also reported that okadaic acid could induce apoptosis [22]. The mechanism that accounts for the differential phenotypes is unclear.

In conclusion, we provide solid evidence that *pr130* plays important roles in regulating cardiac development. Additionally, we highlight the utility of the CRISPR/Cas system in studying targeted-specific genetic modification in zebrafish.

## 4. Experimental Section

### 4.1. Zebrafish Maintenance

Zebrafish and embryos were maintained in a standard environment [23]. Developmental stages were determined according to Kimmel et al. [24]. Zebrafish embryos older than 24 hpf that were used for WISH were treated with 0.003% phenylthiourea to inhibit pigmentation. All animal experiments were approved by the ethics committee from Institute of Hydrobiology, Chinese Academy of Sciences (Approval ID: IHB2013724).

### 4.2. Pr130-Knockout by CRISPR/Cas9 System

Deletion of *ppp2r3a* transcript variant X1 (*pr130*) (XM_003639829) was generated using CRISPR/Cas9 system targeting the first exon of *pr130* gene, where the sequences differ from that of *pr72*. Cas9 target site was designed using ZIFIT Targeter (a web-based online tool) [25]. The targeting site sequences were as follows: 5′-GAAGTTCAGCACAAGTACTG-3′. Cas9 mRNA and gRNA were synthesized using mMESSAGE mMACHINE mRNA transcription synthesis kit (Ambion) and PCR products with forward primer 5′-TAATACGACTCACTATAGGGAAGTTCAGCACAAGTACTGGTTTTAGAGCTAGAAATAGC-3′ and reverse primer 5′-AGCACCGACTCGGTGCCACT-3′ (TranscriptAid T7 High Yield Transcription Kit, Thermo Scientific), respectively [26]. Cas9 mRNA (300 ng/µL) and gRNA (30 ng/µL) were co-injected into one-cell stage WT embryos. To determine mutation efficiency, genomic DNA was extracted from 30 embryos followed by mutation screening using RFLP and Sanger sequencing. The target gene region was amplified with forward primer 5′-CGGCTACTTATCGCATTGTGG-3′ and reverse primer 5′-GGCTGGAGGAGTACTTGATCTC-3′. Remainder embryos were raised to sexual maturity, and F0 and positive F0 adults were mated with WT to obtain the F1 generation. Positive F1 adults were detected as the F0 generation. The positive F1 zebrafish with identical mutation were intercrossed to obtain F2 homozygous mutant offspring and WT offspring. In this experiment, homozygous mutants (M1 and M2) were used for experiments and WT siblings as controls.

### 4.3. RT-PCR and qPCR

Embryonic cardiac tissues and non-cardiac tissues at 72 hpf were isolated from *tg* (*cmlc2: GFP*) zebrafish as described previously [27]. Approximately 300 embryos of transgenic zebrafish with myocardial specific expression of green florescence (*cmlc2: GFP*) were anesthetized. They were collected in 1.5 mL centrifuge tubes, and washed three times with embryo disruption medium (L-15 medium (Invitrogen, Carlsbad, CA, USA) containing 10% fetal bovine serum (Hyclone, Logan, UT, USA). Subsequently, embryos were resuspended in 1.25 mL embryo disruption medium for pumping repeatedly with a 5 mL syringe at a rate of 1 s per syringe motion. After embryonic yolk sac and pericardium had been ruptured, intact green fluorescent protein-positive (GFP+) cardiac tissues were identified and sucked out using a micro-capillary pipette under the fluorescence microscope. Meanwhile, non-cardiac heart tissues were collected in a microtube. Total RNA was extracted using RNeasy^®^ Mini Kit (Qiagen, Valencia, CA, USA) according to manufacturer’s instructions, followed by the reverse transcription using a RevertAid First Strand cDNA Synthesis Kit (Fermentas, ON, Canada). The expression profile of *pr130* was analyzed by RT-PCR and qPCR with forward primer 5′-GAGCGAGAAGTCATGCGTCT-3′ and reverse primer 5′-GACGGTGCAGGTGATAGTGT-3′. The mRNA expression levels were analyzed by qPCR using a CFX96 system (Bio-Rad) with iTaq Universal SYBR Green supermix (Bio-Rad, Hercules, CA, USA). Relative expression was calculated as 2^−^^Δ*C*t^ using β-actin as the endogenous control, and each experiment was performed in triplicate.

### 4.4. PP2A Phosphatase Activity

PP2A activity was determined in adult zebrafish heart tissues using Serine/Threonine Phosphatase Assay System (Promega, V2460, Madison, WI, USA) according to the manufacturer’s instructions. Five adult hearts at 90 dpf were pooled as one sample. Cardiac tissues were homogenized using 1 g of tissue in 3 mL of phosphatase extraction buffer (8 mM imidazole hydrochloride, pH 7.3, 50 mM NaCl, 50 microgram/mL aprotinin, and 10 μM leupeptin, 2 mM EGTA, 5 mM EDTA). Next, 250 μL of tissue lysate was added to a Spin Columns containing Sephadex^®^ G-25 resin to eliminate endogenous phosphate. A standard curve was drawn using phosphate standard dilutions at the concentration of 0, 100, 200, 500, 1000, and 2000 pmol. Next, 10 μL of PPase-2A 5× reaction buffer (250 mM imidazole, pH 7.2, 1 mM EGTA, 0.1% β-mercaptoethanol, 0.5 mg/mL BSA) and 5 μL of 1 mM phosphopeptide were plated in supplied 1/2-area flat-bottom 96-well plate. The 96-well plate was placed at room temperature for 3 min. Add next, 35 µL of tissue lysate was added to the 96-wells and incubated for 30 min at room temperature. The reaction was stopped by adding 50 μL of molydate dye/additive mixture to the 96-well plate containing the samples and the phosphate standard dilutions. The 96-well plate was incubated at room temperature for 15 min. Free phosphate generated from a phosphor-peptide was quantified by measuring the absorbance of the molybdate: malachite green: phosphate complex at 600 nm. Each experiment was performed with three replications.

### 4.5. Western Blot Analysis

Total protein was extracted from five dpf embryos with Radioimmunoprecipitation assay (RIPA) lysis buffer, then denatured at 95 °C for 10 min. About 60 μg of total protein was resolved by sodium dodecyl sulfate polyacrylamide gel electrophoresis (SDS-PAGE) on 10% gel and then transferred to a polyvinyl difluoride (PVDF) membrane (Millipore, Hayward, CA, USA) onto Mini-PROTEAN Tetra (Bio-Rad, Hercules, CA, USA) that was blocked in Tris-buffered saline (TBS) containing 5% bovine serum albumin (BSA). After incubated overnight at 4 °C with the following primary antibodies: rabbit anti-PPP2R3A (Abcam, ab126195) and mouse anti-GAPDH (proteintech, 60004-1-Ig), the PVDF membrane was incubated with the secondary antibodies (proteintech, SA 00001-1, SA 00001-2) for 2 h. The signal was visualized using chemiluminescence (ImageQuant LAS 4000mini).

### 4.6. WISH

We carried out WISH as described previously [28,29]. DIG-labeled antisense RNA probe of zebrafish *cmlc2* was synthesized and applied for WISH as described previously. The *cmlc2* probe was amplified from cDNA with forward primer CAGACCAACAGCAAAGCAG and reverse primer TAATACGACTCACTATAGGGACTGCAACTGAGTATGAAGTTTATTATAG.

### 4.7. Histological Studies

H&E staining of myocardium of WT and mutant zebrafish at 90 dpf was performed as previously described [30]. Briefly, heart tissues were fixed in 4% paraformaldehyde and embedded in paraffin wax. Zebrafish hearts were observed in serial sections using an Olympus microscope. The number of cardiomyocytes in each of four chosen fields per fish (16 fish per group) at a magnification of 1000× was calculated using Image J software and compared among WT, M1, and M2.

### 4.8. Quantification of the Embryonic Heart Function

The physiological function of 48-hpf embryonic hearts was analyzed by semi-automated optical heartbeat analysis under an Olympus IX71 microscope with HC Image software [31,32,33].

### 4.9. Transmission Electron Microscopy

The ultrastructure of adult zebrafish hearts at 90 dpf was observed using transmission electron microscopy [34]. Heart-tissues were fixed in 2% glutaraldehyde, embedded in Epon 812, and cut into 100-nm-thick slices using UC7 ultramicrotome (Leica, Heerbrugg, Switzerland), then stained with uranyl acetate and lead citrate. Finally, the images of myocardial ultra-structure were captured by a Hitachi-7700 transmission electron microscope (Hitachi, Tokyo, Japan).

### 4.10. Tunel Staining

Tunel assay was used to detect apoptotic nuclei of adult zebrafish heart sections using in situ a cell death detection kit (Roche, Pleasanton, CA, USA), following the instructions of the manufacturer [35]. Apoptotic cells were determined by manual counting of Diaminobenzidine (DAB) positive nuclei (brown). TUNEL-positive cells were counted and analyzed in six fields of each zebrafish by two independent investigators.

### 4.11. Statistical Analysis

The differences in quantitative and qualitative variables between different groups were assessed by Student *t*-test (or ANOVA test) and Pearson χ^2^ test, respectively. *p* < 0.05 were considered statistically significant. All statistical analyses were performed by SPSS20.0.
